# Bacteroides fragilis Abscess Associated With Fat Necrosis Following Breast Augmentation With Autologous Fat Grafting: A Case Report With Review of the Literature

**DOI:** 10.7759/cureus.116653

**Published:** 2026-09-21

**Authors:** Nadiia Redmann, Shrikant K Goyal, Negin Niknam, Harika Kalangi, Assaf Holtzman

**Affiliations:** 1 Infectious Diseases, Donald & Barbara Zucker School of Medicine at Hofstra/Northwell, Manhasset, USA; 2 Biochemistry, Stony Brook University, Stony Brook, USA

**Keywords:** autologous fat grafting, bacteroides fragilis, cosmetic breast augmentation, fat necrosis, liposuction complications

## Abstract

Autologous fat grafting (AFG) is a popular breast augmentation procedure characterized by high patient satisfaction and low complication rates. Infectious complications are uncommon with this technique, and gram-positive organisms are the predominant pathogens responsible for most cases. We report a rare case of abscess formation due to *Bacteroides fragilis *infection occurring in the setting of presumed fat necrosis following AFG, as indicated by ultrasound imaging and plastic surgery evaluation.

A 52-year-old woman developed a postoperative infection after AFG and abdominal liposuction despite receiving ciprofloxacin for prevention of infectious complications. Initial imaging suggested postsurgical changes, and she was treated with oral amoxicillin-clavulanate. She re-presented two weeks later with fever and worsening bilateral breast pain; repeat imaging revealed bilateral rim-enhancing fluid collections. Aspiration cultures grew *Bacteroides fragilis*. The patient initially improved with intravenous (IV) antibiotics followed by oral metronidazole; cefadroxil was additionally prescribed to provide empiric coverage for gram-positive organisms, given prior antibiotic exposure that may have affected culture results. However, the patient subsequently developed recurrent disease and ultimately required incision and drainage.

This case highlights a rare anaerobic infection after AFG and emphasizes the need for early recognition and appropriate antimicrobial coverage.

## Introduction

Autologous fat grafting (AFG) is a frequently performed cosmetic procedure for breast augmentation in the United States. Its popularity is largely attributable to high patient satisfaction and a relatively low incidence of long-term complications. Additional benefits include the use of autologous tissue for implantation and the simultaneous removal of unwanted fat from other areas of the body. Although generally considered safe, AFG has been associated with complications such as fat necrosis, infection, oil cyst formation, and hematoma, with reported rates varying according to surgical technique, fat-processing methods, and patient-related factors. Fat necrosis is one of the most common complications following AFG and is primarily caused by inadequate revascularization of transplanted adipose tissue. The resulting ischemia and hypoxia lead to adipocyte death, triggering a sterile inflammatory response characterized by macrophage infiltration, lipid release, fibrosis, and, in some cases, calcification. Larger graft volumes and poor vascular integration may further increase the risk of fat necrosis [1,2].

The clinical presentation of fat necrosis may include a palpable breast mass, pain, inflammatory skin changes, drainage, or progressive breast asymmetry. Because these findings are nonspecific, the differential diagnosis includes seroma, hematoma, infected oil cyst, cellulitis or abscess, granulomatous mastitis, postoperative inflammatory changes, and breast carcinoma. Radiologic evaluation with ultrasound, mammography, or magnetic resonance imaging is often necessary because fat necrosis may demonstrate features that overlap with those of malignancy. When the diagnosis remains uncertain, image-guided biopsy and histopathologic evaluation may be required. Once fat necrosis has been confirmed, asymptomatic lesions can often be managed with observation and routine imaging follow-up. However, persistent lesions or those causing pain or breast deformity may require surgical excision. Oil cysts containing liquefied fat may be treated with needle aspiration to relieve symptoms. For solid masses or contour deformities, treatment is guided by the anticipated post-excision defect and may range from simple excision to extensive debridement and reconstructive surgery for larger areas of necrosis [3].

Multiple systematic reviews have demonstrated that AFG for breast augmentation is associated with a low incidence of serious adverse events. Infectious complications are particularly uncommon, with reported rates ranging from approximately 0.6% to 2% [4-7]. When infection occurs, it is most commonly caused by gram-positive organisms, including *Staphylococcus aureus* and coagulase-negative *Staphylococcus *species. Gram-negative organisms, anaerobes, and nontuberculous mycobacteria are reported less frequently. Although anaerobes are rarely identified as the sole causative organisms of infections following AFG, they may be part of polymicrobial infections, particularly in the presence of deep abscesses or underlying tissue necrosis [8].

The occurrence of a *Bacteroides fragilis* breast abscess associated with fat necrosis after autologous breast augmentation is exceedingly rare. Because the clinical presentation may resemble more common postoperative complications or breast malignancy, early recognition and appropriate microbiologic evaluation are essential. We report a rare case of *Bacteroides fragilis* breast abscess associated with presumed fat necrosis following AFG breast augmentation, based on ultrasound findings and plastic surgery evaluation.

## Case presentation

A 52-year-old woman with no significant past medical history and no home medications presented to the emergency department with fever, left breast tenderness, and abdominal firmness approximately two weeks after undergoing bilateral breast augmentation with AFG following abdominal liposuction. Her social history was negative for tobacco, alcohol, and illicit drug use. She worked from home and had no pets. She had completed a 10-day course of ciprofloxacin following the procedure for infection prevention.

A computed tomography (CT) scan of the chest, abdomen, and pelvis revealed ill-marginated soft tissue densities in the deep retroareolar regions of both breasts, as well as soft tissue infiltration in the subcutaneous fat of the anterior abdominal wall, consistent with recent liposuction and small associated fluid collections. She was evaluated by the surgical team and discharged home on amoxicillin-clavulanate for seven days. The changes on the CT scan were considered postoperative changes.

Fifteen days later, the patient returned to the emergency department with a fever of 101.8°F, chills, and worsening bilateral breast pain associated with heaviness and fullness. On examination, the patient was in mild distress due to breast pain. There was tenderness and firmness on palpation of both breasts, although the postsurgical incisions were healed. The abdominal examination revealed no tenderness or distention. A scar from a previous cesarean section was observed. Blood work on presentation was significant for mild leukocytosis and reactive thrombocytosis, as shown in Table 1. Blood cultures were obtained and remained negative. 

**Table 1:** Laboratory findings on second presentation.

Test	Result	Reference range
WBC count	11.38 × 10^3^/µL	3.80-10.50 × 10^3^/µL
Auto neutrophil	84.0 %	43.0-77.0%
Auto lymphocyte	9.0%	13.0-44.0%
Auto monocyte	4.7%	2.0-14.0%
Auto eosinophil	1.5%	0.0-6.0%
Auto basophil	0.4%	0.0-2.0%
Hemoglobin	9.2 g/dL	11.5-15.5 g/dL
Platelet count	480 × 10^3^/µL	150-400 × 10^3^/µL

Ultrasound of the bilateral breasts showed the following: the breast parenchyma demonstrated a heterogeneous background echotexture (mixed fatty and fibroglandular). Left breast: deep heterogeneous but predominantly mildly echogenic mass measuring 7 × 3 × 8.3 cm, likely representing a fluid collection (Figure 1). Right breast: a deep ovoid mildly hypoechoic mass measuring 5.4 × 2.1 × 6.1 cm, likely representing a fluid collection; and an echogenic nodule measuring 2.5 × 1.4 × 2.3 cm with a surrounding hypoechoic rim, probably representing an area of fat necrosis (Figure 2).

**Figure 1:**
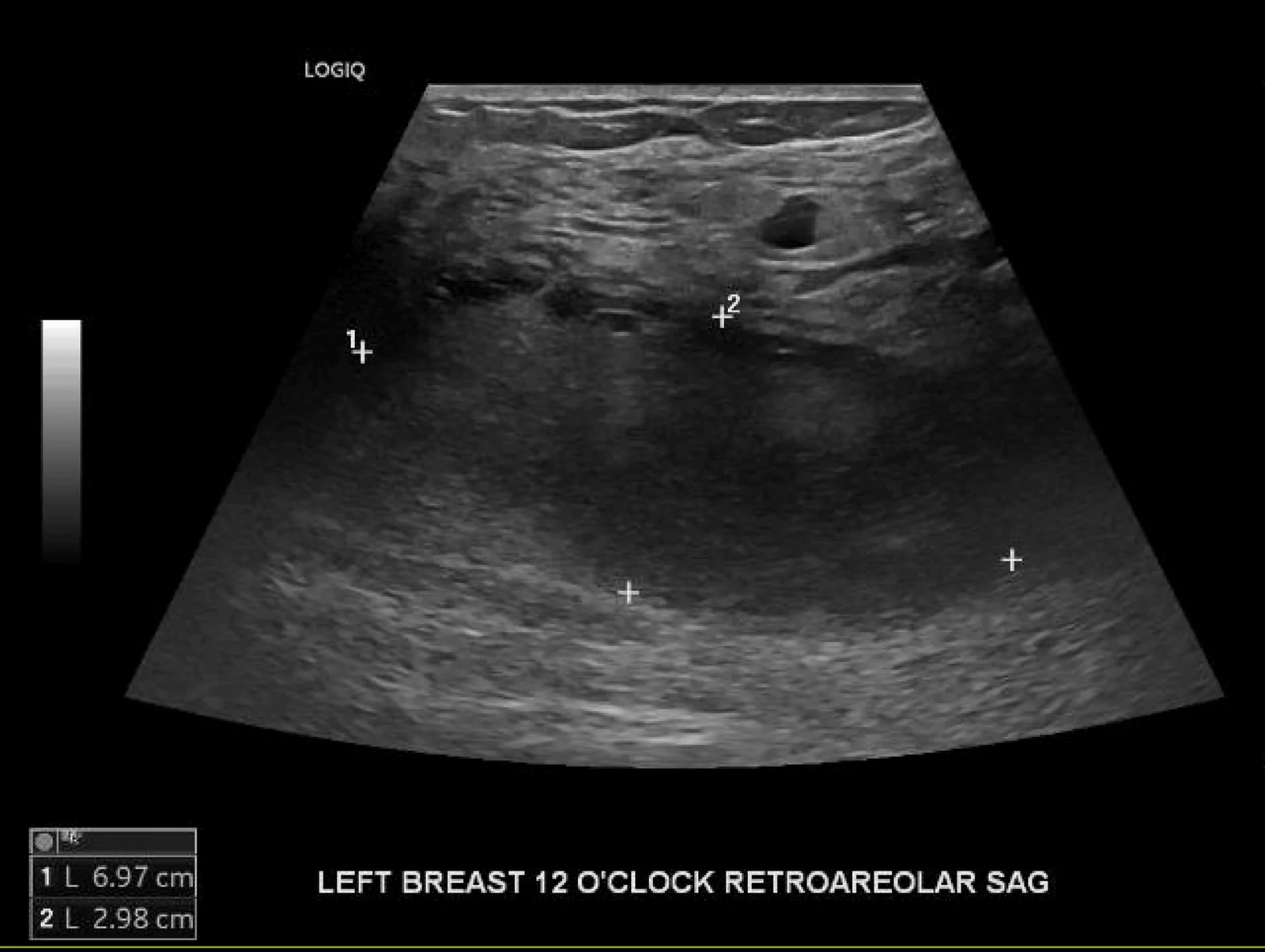
Ultrasound of the left breast. Sonographic evaluation of the left breast was performed in its entirety, including each of the four quadrants and the retroareolar region, in a clockwise fashion. The breast parenchyma demonstrated a heterogeneous background echotexture (mixed fatty and fibroglandular tissue). At the 12:00 position in the retroareolar region, a deep heterogeneous but predominantly mildly echogenic mass measuring 7 x 3 x 8.3 cm, likely representing a fluid collection.

**Figure 2:**
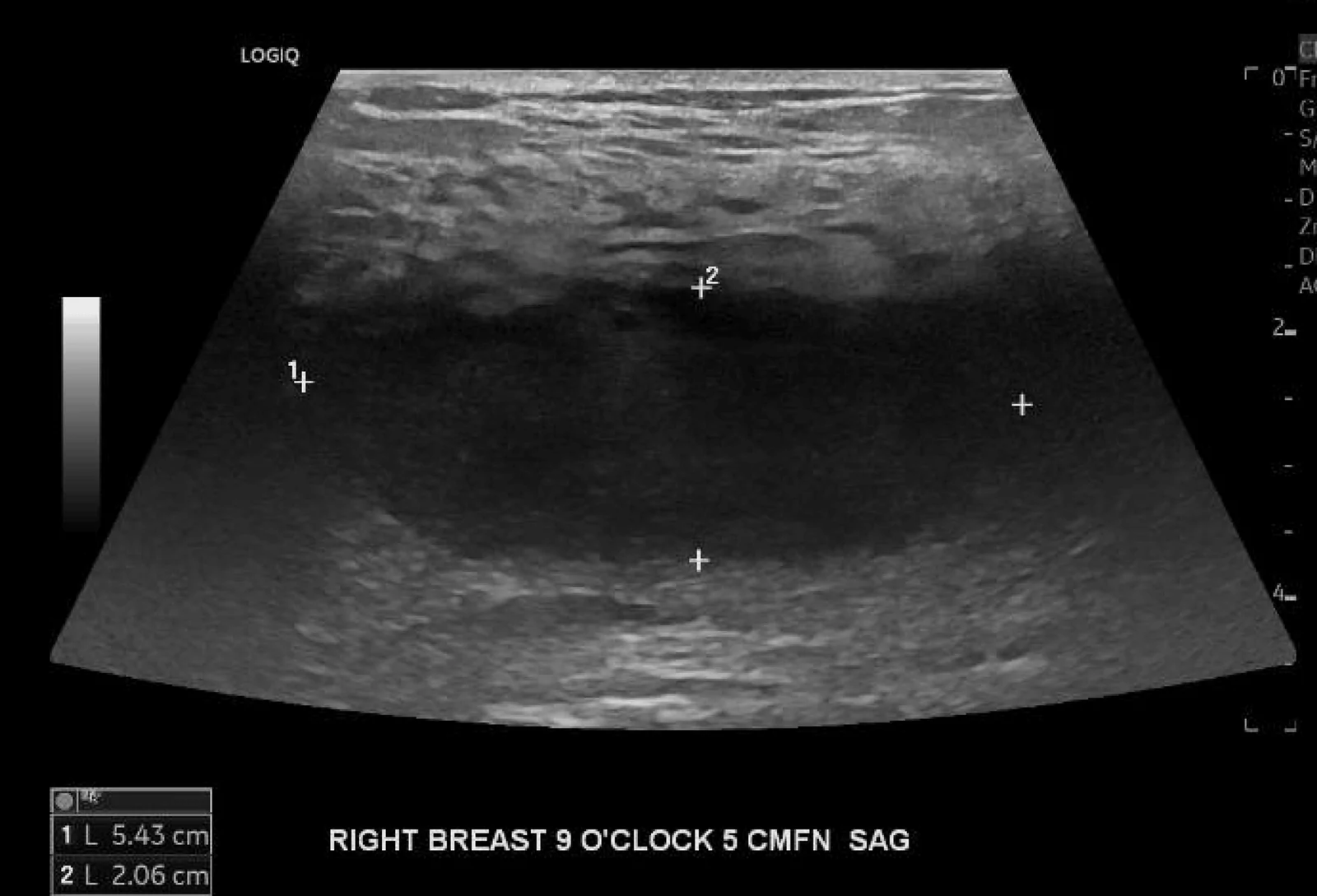
Ultrasound of the right breast. Sonographic evaluation of the right breast was performed in its entirety, including each of the four quadrants and the retroareolar region, in a clockwise fashion. The breast parenchyma demonstrated heterogeneous background echotexture (mixed fatty and fibroglandular tissue). At the 9:00 position, 5 cm from the nipple, there was a deep ovoid mildly hypoechoic mass measuring 5.4 x 2.1 x 6.1 cm, likely representing a fluid collection.

A CT scan of the chest, abdomen, and pelvis with intravenous contrast revealed bilateral rim-enhancing breast fluid collections containing locules of fat density, measuring 9.9 × 3.6 × 7.6 cm on the right and 11.2 × 4.1 × 8.2 cm on the left. Associated increased soft tissue density of the surrounding breast parenchyma was likely reactive. Mild bilateral axillary lymphadenopathy was also present. Postsurgical changes were noted within the abdominal wall. Additionally, a stable periumbilical fluid collection measuring 1.5 × 1.5 cm was identified.

The patient was started on empiric intravenous cefepime and vancomycin. She was seen by a plastic surgeon in the emergency room, where aspiration of fluid from the left breast was performed. The next day, an interventional radiologist performed percutaneous aspiration of the bilateral fluid collections, yielding 2 mL of yellowish fluid from each side. The limited volume of aspirated fluid may have been attributable to tissue densities and areas of presumed fat necrosis that appeared as fluid collections on imaging. Cultures from both samples were positive for *Bacteroides fragilis*, and one of the two samples also grew *Bacteroides thetaiotaomicron *group organisms. Culture performed on the aspirate collected in the emergency room also grew *Bacteroides fragilis*. Fungal and acid-fast bacilli cultures were negative. A decision was made to treat the patient conservatively. Her fevers subsequently resolved, and she was discharged on a 14-day course of definitive treatment with oral metronidazole 500 mg three times daily. Cefadroxil was additionally prescribed to provide empiric coverage for gram-positive organisms, given her prior exposure to amoxicillin-clavulanate (Augmentin), which may have affected the culture results.

Three weeks after discharge, the patient re-presented to the emergency room due to worsening left breast pain and persistent night sweats. Plastic surgery performed incision and drainage of the abscess, after which the patient reported significant symptomatic improvement. Abscess cultures remained negative, and she was subsequently discharged on a course of cefadroxil and metronidazole.

## Discussion

Fat grafting to the breasts has developed in recent years and now serves a variety of roles in aesthetic breast surgery. It is utilized for both reconstructive and cosmetic reasons [6]. AFG begins with harvesting fat through liposuction from areas like the thighs, abdomen, or flanks. After careful processing, this fat is grafted to the recipient site for reconstruction. In recent years, AFG has gained remarkable traction in breast reconstruction because it combines two powerful advantages: using the patient’s own tissue and offering a minimally invasive approach, delivering natural, lasting results. Reconstructive uses include complementing breast reconstruction, correcting contours after partial mastectomy, treating radiation injury deformities, and potentially achieving complete breast restoration after mastectomy. Cosmetic uses involve primary augmentation without implants, restoring volume after implant removal, and refining implants to conceal rippling [6,9].

AFG (natural fat transfer for breast augmentation) is generally believed to have fewer complications than traditional breast implants or myocutaneous flap procedures [6]. Complications following the procedure can be categorized by their onset. Early complications, occurring within four weeks, include induration, fat necrosis, and surgical site infections. Late complications, which manifest after four weeks, involve liponecrosis cysts, further fat necrosis or resorption, calcification, and unsatisfactory aesthetic results [10]. Groen et al. analyzed AFG in 3,565 patients for cosmetic and reconstructive purposes, reporting a 17.2% complication rate, with induration being the most common finding. Infection of the breast was seen and treated with oral antibiotics, drainage, and/or ice packing in 0.9% of the cases, and donor site infection was seen in 0.6% of the cases [4].

In another systematic review, Largo and colleagues analyzed 1,453 patients with a mean follow-up period of 16.3 months (1-156 months). Major complications, defined as those requiring surgical management or hospitalization, were seen in 88 patients (6.1%). Infections requiring surgery were observed in 35 patients (2.4%). They noted that technical factors, including the use of bolus fat injections rather than meticulous fat distribution, may contribute to complications following AFG [7].

Ørholt and colleagues, in their review of 22 studies with 2,073 patients, reported that 2.1% of patients experienced some type of complication. Infectious complications following breast augmentation were usually infrequent, occurring in approximately 0.6% of patients. Their study included mostly patients undergoing cosmetic breast augmentation. Higher rates of infection and other complications have been reported in reviews, including patients undergoing both reconstructive and cosmetic breast augmentation by AFG [5].

Breast infections after AFG are uncommon and generally result from contamination with normal skin flora, including Staphylococcus aureus and coagulase-negative staphylococci. Gram-negative organisms and anaerobes are also reported. Unlike in our case, anaerobes are rarely the sole cause of infection, usually causing polymicrobial infections, particularly in deep abscesses or underlying necrosis [11].

Although uncommon, nontuberculous mycobacterial infections have also been reported. Over the past two years, Tan, Liu-Chang et al. treated five patients who developed nontuberculous mycobacterial infections after AFG performed by four different surgeons [8]. Another rare case described a patient who developed a Corynebacterium bovis infection after receiving a second injection of cryopreserved fat, which was suspected to be the source of contamination [12].

Despite the low rate of infectious complications, rare cases of devastating infections, including necrotizing fasciitis associated with fat grafting, have been described. Chong Han Pek et al. reported a patient who required urgent surgical and antibiotic treatment. After multiple debridements and washouts, bacterial cultures grew *Proteus mirabilis*, *Enterobacter aerogenes*, *Enterococcus faecalis*, and *Bacteroides fragilis* [13]. Two other case reports described patients who presented with sepsis and multiple breast abscesses following fat grafting, with cultures obtained after drainage growing *Escherichia coli*, alpha-*Streptococcus*, group A *Streptococcus*, *Peptostreptococcus*, and the anaerobic bacterium *Bacteroides fragilis* [14,15].

We report a rare case involving an unusual isolate consisting solely of *Bacteroides* species. *Bacteroides* are obligate anaerobic, gram-negative bacilli. *Bacteroides fragilis* is a component of the normal human colonic microbiota but can cause clinically significant infection when the mucosal surface is disrupted by inflammation, trauma, or surgery, leading to spread into the bloodstream or surrounding tissues [15].

The mechanism of infection after AFG usually includes the introduction of skin bacteria into the wound during liposuction or injection, despite sterile skin preparation. However, in our patient, given the culture result, potential modes of infection are either contaminated fat processing or superinfection after fat necrosis.

The harvested fat is often centrifuged, washed, and transferred between containers. Each step is a potential point of contamination if not performed in a completely closed and sterile system. The presence of necrotic fat graft tissue creates an ideal anaerobic environment for *Bacteroides *to flourish. Most of these infections are polymicrobial, unlike in our patient, whose cultures grew only *Bacteroides* spp. There is a possibility, though, that given prior antibiotic use, the culture result might have been affected, although it is much harder to grow anaerobes than other forms of bacteria.

In the context of AFG, infection with *Bacteroides fragilis* is uncommon but can occur because injected fat may undergo necrosis, as in our case, creating low-oxygen conditions that favor anaerobic growth. *Bacteroides fragilis* is generally susceptible to metronidazole, carbapenems, and beta-lactam/beta-lactamase inhibitor combinations. However, antimicrobial susceptibility patterns may vary by geographic region and over time, with increasing resistance reported to agents such as clindamycin and moxifloxacin [16]. Therefore, antimicrobial selection should be guided by local antibiogram data and susceptibility testing when available. In our case, the isolate was successfully treated with metronidazole-based therapy.

Our patient did well after incision and drainage in combination with antibiotics, highlighting the need for appropriate source control to achieve optimal outcomes.

## Conclusions

Our case describes a rare *Bacteroides fragilis* infection with fat necrosis following AFG breast augmentation. Although anaerobic infections following AFG are uncommon, they should be considered in patients with suspected fat necrosis and associated infection. While a causal relationship cannot be established, necrotic tissue may create a relatively low-oxygen environment that can support the growth of anaerobic organisms.
